# MiRNA-766-3p inhibits gastric cancer via targeting COL1A1 and regulating PI3K/AKT signaling pathway

**DOI:** 10.7150/jca.90321

**Published:** 2024-01-01

**Authors:** Yujie Ding, Mengyuan Zhang, Sheng Hu, Caiyun Zhang, Yue Zhou, Ming Han, Jingjing Li, Fulong Li, Hongmei Ni, Shengquan Fang, Qilong Chen

**Affiliations:** 1 School of Traditional Chinese Medicine, Shanghai University of Traditional Chinese Medicine, Shanghai 201203, China.; 2 Department of Gastroenterology, Yueyang Hospital of Integrated Traditional Chinese and Western Medicine, Shanghai University of Traditional Chinese Medicine, Shanghai 200437, China.; 3 Central Laboratory, Shanghai Skin Disease Hospital, School of Medicine, Tongji University, Shanghai 200443, China.; 4 Shanghai Institute of Stem Cell Research and Clinical Translation, Shanghai 200120, China.

**Keywords:** MiRNA-766-3p, COL1A1, PI3K/AKT signal pathway, Gastric cancer.

## Abstract

**Objective** MiRNA-766-3p has been shown to be associated with a variety of cancers. However, few studies have been done in gastric cancer (GC). This study explores the mechanism of miR-766-3p in GC.

**Methods** The potential targets of microRNA (miRNA) were predicted using Tarbase and Targetscan databases. The results are intersected with differential genes (DEGs) (fold change > 1.5, P < 0.05) in gastric cancer to obtain potential core targets. The hub targets screened by constructing PPI networks (degree > 5, expression > 0.5). Validating the differential expression and expression in immunohistochemistry of these targets through the database. And the binding sites between miRNAs and mRNAs were verified using dual-luciferase Assay. Finally, qRT-PCR and Western Blot experiments were conducted to validate the hub targets and signal pathways.

**Results** The potential hub targets from the PPI network were THBS2, COL1A1, FGG, FGB, and PLAU. Combining database, luciferase Assay and experimental validation, miR-766-3p can sponge COL1A1 and it plays the most important role in gastric cancer progression. In GC, COL1A1 was upregulated and the enrichment analysis revealed that COL1A1 regulates PI3K/AKT signal pathway, and AKT is also highly expressed in gastric cancer.

**Conclusion** The miR-766-3p can inhibit the progression of gastric cancer by targeting COL1A1 and regulating the PI3K/AKT signal pathway. It could be a potential therapy option for the GC.

## Introduction

Gastric cancer (GC) remains a major cancer worldwide, accounting for over one million new cases in 2020 and an anticipated 769,000 deaths, ranking fourth for mortality and fifth for incidence globally 1. Surgery and chemotherapy remain the most effective treatment modality and gene therapy have seen significant advancements in the past decade 2. However, due to the low rate of early detection and diagnosis, many patients with gastric cancer have very poor survival, and the overall 5-year survival rate of GC patients is less than 50% in China 3,4. Gastric cancer development is a complicated process that involves many genetic and epigenetic changes in oncogenes, tumor suppressor genes, DNA repair genes, cell cycle regulators, and signaling molecules 5. Therefore, it is crucial to elucidate the pathogenesis of gastric cancer by identifying novel targets for treatment.

MicroRNA (miRNA), 18-22 nucleotides in length, is a class of small noncoding RNA molecules with highly conserved. It is generally considered that miRNAs influence gene expression at the transcriptional level via binding to mRNA 6. Much evidence demonstrates that miRNAs correlate with various tumors, such as breast cancer, lung cancer and ovarian cancer 7-9. It contributes to cancer through a nuclear function that affects gene transcription and epigenetic states 10. Importantly, miRNA is also associated with gastric cancer progression. MiR-34a inhibits the proliferation and invasion of gastric cancer by regulating PDL1^11^. MiR-1262 inhibits gastric cardia cancer by targeting the oncogene ULK1^12^. MiR-542-3p suppresses cell proliferation by targeting the oncogene astrocyte-elevated gene-1^13^. This evidence shows that miRNAs involved in gastric cancer progression.

Our previous work demonstrated that miR-766-3p plays a kernel role in GC by identifying the differentially expressed (DE) miRNAs of GC 14. The study shows that the miR-766-3p contributes to anti-inflammatory responses and stops the inflammatory carcinoma transformation by inhibiting NF-κB signaling indirectly 15. It has also been demonstrated to target a variety of oncogenes, such as hepatocellular carcinoma, colorectal cancer, renal cell carcinoma and thyroid carcinoma. The miR-766-3p/FOSL2 axis plays an oncogenic role in hepatocellular carcinoma 16. It inhibits proliferation in colorectal cancer cells via the PI3K/AKT pathway when HNF4G is down-regulated 17. MiR-766-3p also targets and inhibits SF2 expression and promotes the proliferation of renal cell carcinoma cells 18. Circ_0059354 accelerates the growth of papillary thyroid cancer by increasing ARFGEF1 levels via miR-766-3p sponging 19. These studies have shown that miR-766-3p plays an important role in cancer. However, few studies have been conducted to explore the mechanisms of miR-766-3p in gastric cancer. In this paper, the core targets and signal pathway of miR-766-3p in gastric cancer was further analyzed. We identified COL1A1 as the core target of miR-766-3p through database prediction and screening. The binding sites between miR-766-3p and COL1A1 were verified using dual luciferase Assay. Biological Functional Analysis was used and found that COL1A1 correlates with PI3K/AKT signal pathway. Experiments were used to validate the conclusions of the data analyses.

## Materials and methods

### Patients and samples

From July 2022 to May 2023, a total of 60 clinical tissue samples (30 tumor samples and 30 adjacent normal samples) were collected from patients at Yueyang Hospital of Integrated Traditional Chinese and Western (Table 1).

Inclusion criteria: (1) Clinical specimens were confirmed to be gastric cancer by histopathological examination and there was at least one solid or measurable extra-gastric lesion; (2) 18 years old ≤ 85 years old; (3) Eastern Cooperative Oncology Group (ECOG) score of 0-2; (4) Patients or authorized relatives of the patients signed the informed consent before enrolment. Exclusion criteria: (1) patients who have received radiotherapy or chemotherapy; (2) history of other tumors within 5 years; (3) patients in pregnancy or lactation; (4) combined organ failure or other serious diseases; (5) combined neurological or psychiatric history. And paraneoplastic tissue is taken from at least 4 cm away from the tumor lesion. All tissue samples were immediately frozen and preserved in liquid nitrogen until further use. Samples were then kept at -80°C for RNA protein extraction.

### MiR766-3p targets prediction and enrichment analysis

The miRNA target genes were predicted using two databases involving TarBase (http://www.diana.pcbi.upenn.edu/tarbase) (v8.0) and TargetScan (http://www.targetscan.org/vert_72/), which contained the largest collection of manually curated experimental data. The signal pathway of the hub target was enriched using DAVID (https://david.ncifcrf.gov) online.

### Differentially expressed genes (DEGs) identification

The mRNA dataset (GSE118916) of gastric cancer was obtained from the GEO database (http://www.diana.pcbi.upenn.edu/tarbase), which includes 30 GC tissue samples and 30 normal samples. The mRNA levels of all samples were standardized using DESeq software, and the difference significance test of reads was performed using NB (Negative binomial distribution). R package was used to identify the differentially expressed genes (DEGs) between the Tumor Group and Normal Group (fold change > 1.5, P < 0.05).

### Protein-protein interaction (PPI) network construction

The DEGs were used to construct a PPI network by the STRING (v11.5), and CytoHubba clusters were used to gain hub genes. The determinate nodes were considered as potential hub mRNAs, and they will be further validated in database and experiments.

### Core genes validated by databases

Data from the TCGA (The Cancer Genome Atlas) and the GTEx (Genotype-Tissue Expression) projects were used to conduct expression and survival analyses for possible indicators. The Human Protein Atlas (HPA) database (https://www.proteinatlas.org) was then used to obtain core proteins immunohistochemistry results between stomach glandular cells and tumor cells.

### Dual-luciferase reporter assay

The binding sites of miR-766-3p and COL1A1 were calculated and predicted using miRanda (v3.3). The wild-type and mutant plasmids of COL1A1 (psiCHECK-2-WT-COL1A1 3′UTR, psiCHECK-2-MUT-COL1A1 3′UTR) were constructed and provided by Yilaibo (http://www.shyilaibo.com). Luciferase activity was assessed with a dual luciferase kit (E1901; Promega, USA) 48 h after co-transfection of each plasmid with miR-NC or miR-766-3p.

### qRT-PCR validation

Total RNA was isolated using FreeZol Reagent 200rxns (R711-01, Vazyme). Reverse transcription was operated using a miRNA 1st Strand cDNA Synthesis Kit (MR101-01, Vazyme), and HiScript II Q RT Super Mix for qPCR (R223-01, Vazyme). Then, using the miRNA Universal SYBR PCR MasterMix (MQ101-01, Vazyme) and ChamQ SYBR qPCR Master Mix (Q321-02, Vazyme), the levels of miRNA and mRNA expression were determined. GAPDH and U6 were employed as endogenous controls for mRNA and miRNA expression levels, respectively. Finally, the relative RNA expression levels were determined using the 2^-ΔΔCt^ technique. Table 2 displays the primer sequences.

### Western blot

RAPI reagents (epizyme) were used to extract total proteins. Protein samples were loaded and separated by 7.5% SDS-PAGE before being transferred to NC membranes and blocked at room temperature for 1 hour. The membranes were washed five times with TBST before being incubated overnight at 4°C with anti-COL1A1 (1:1000, 72026T, CST) and anti-GAPDH (1:1000, 5174S, CST). The membranes were then treated for 1 hour at room temperature with horseradish peroxidase (HRP) conjugated second antibodies (Proteintech). In a dark chamber, the films were developed and fixed with an ECL solution. Image J 1.2.4 (NIH, USA) was used to semi-quantify protein expression.

### Statistical analyses

All variables are provided as mean SD and statistical analysis was conducted using the SPSS 23.0 program (IBM Analytics). The groups were compared using two-tailed Student's t-tests, and a P value of 0.05 was regarded as statistically significant. Plotting was done using GraphPad Prism 8 software (GraphPad Software Inc., San Diego, CA, USA).

## Results

### Potential targets of miR-766-3p related to gastric cancer

**Figure 1:**
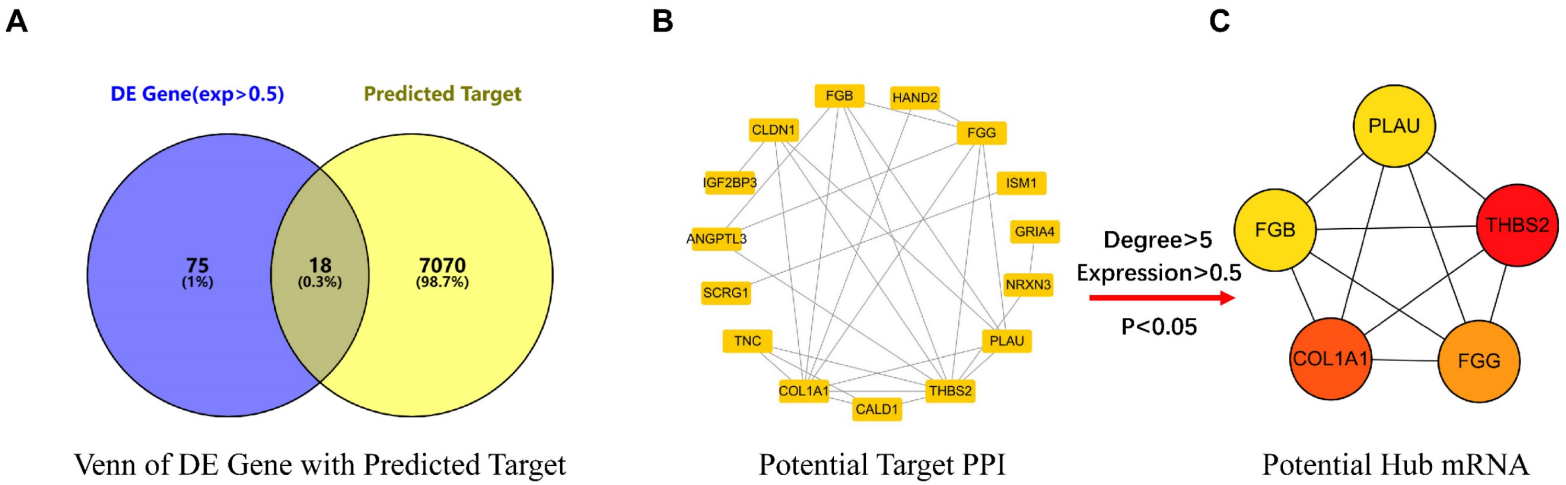
Potential mRNA of miR-766-3p related to gastric cancer screening. (A) The Venn diagram of DE Gene and predicted target taking the intersection; (B) The 18 DE mRNAs from the Venn diagram were used to build the PPI network; (C) The 5 core DE mRNAs were screened from PPI network.

Using the prediction programs, the miR-766-3p target genes were predicted. As a result, a total of 7088 targets were collected from Tarbase and TargetScan. Using the R package, 93 DE mRNAs were accessed by analyzing 30 gastric cancer clinical microarray data from the GEO database. In this work, we intersected the 93 DE mRNAs with 7088 predicted targets and required target expression abundance greater than 0.5. Following the preceding stages, 18 candidate targets were obtained eventually (Fig. 1A).

### PPI network construction and hub mRNAs screening

Based on the STRING database, the PPI network was constructed by 18 DE mRNAs (Fig. 1B). We employed the CytoHubba cluster (Top5) to screen the key mRNA of the PPI, and five core targets were eventually selected (THBS2, COL1A, FGG, FGB, PLAU) (Fig. 1C, Table 3).

### Validation core markers based on TCGA and HPA database

**Figure 2:**
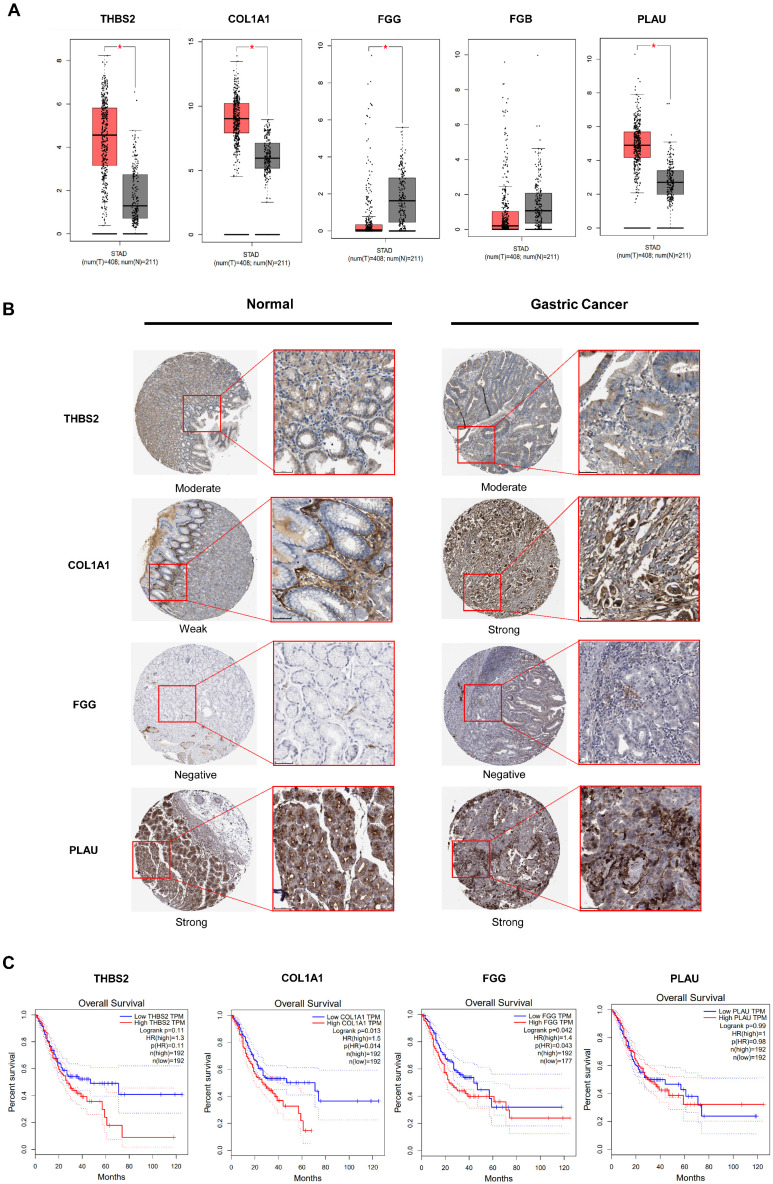
GEPIA and TCGA datasets were used for expression and survival analysis for prospective targets. (A) Based on the TCGA database, the expression of 5 potential markers was estimated (Tumor group, n = 408; Normal group, n = 211); (B) The immunohistochemistry results of the THBS2, COL1A1, FGG, PLAU between human gastric tumor and normal tissues; (C) Survival analysis was used to determine the prognostic significance of targets in gastric cancer patients.

Gastric cancer (n = 408) and normal (n = 211) expression of five key genes was assessed using GEPIA, respectively. The result revealed that the expression of THBS2, COL1A, FGG, and PLAU was considerably different (Fig. 2A). To further analyze the levels of 4 targets, we used the HPA database to validate the tissue expression in immuno-histochemistry. Only one target showed variations in expression between tumor and normal tissues (Fig. 2B). The COL1A1 was negative in normal tissues but strongly expressed in tumors, and immuno-histochemistry revealed no differences in the expression of other targets. In addition, we investigated the prognostic values of COL1A1 in gastric cancer patients based on the overall survival (OS) calculation. According to the findings, the high mRNA levels of COL1A1 have statically significant (P = 0.014) of OS in gastric cancer patients (Fig. 2C). Other targets had no impact on overall survival. Based on the confirmation of the data presented above, it is possible to conclude that COL1A1 is very significant to gastric cancer and is an essential target for miR-766-3p.

### Dual-luciferase reporter assay

**Figure 3:**
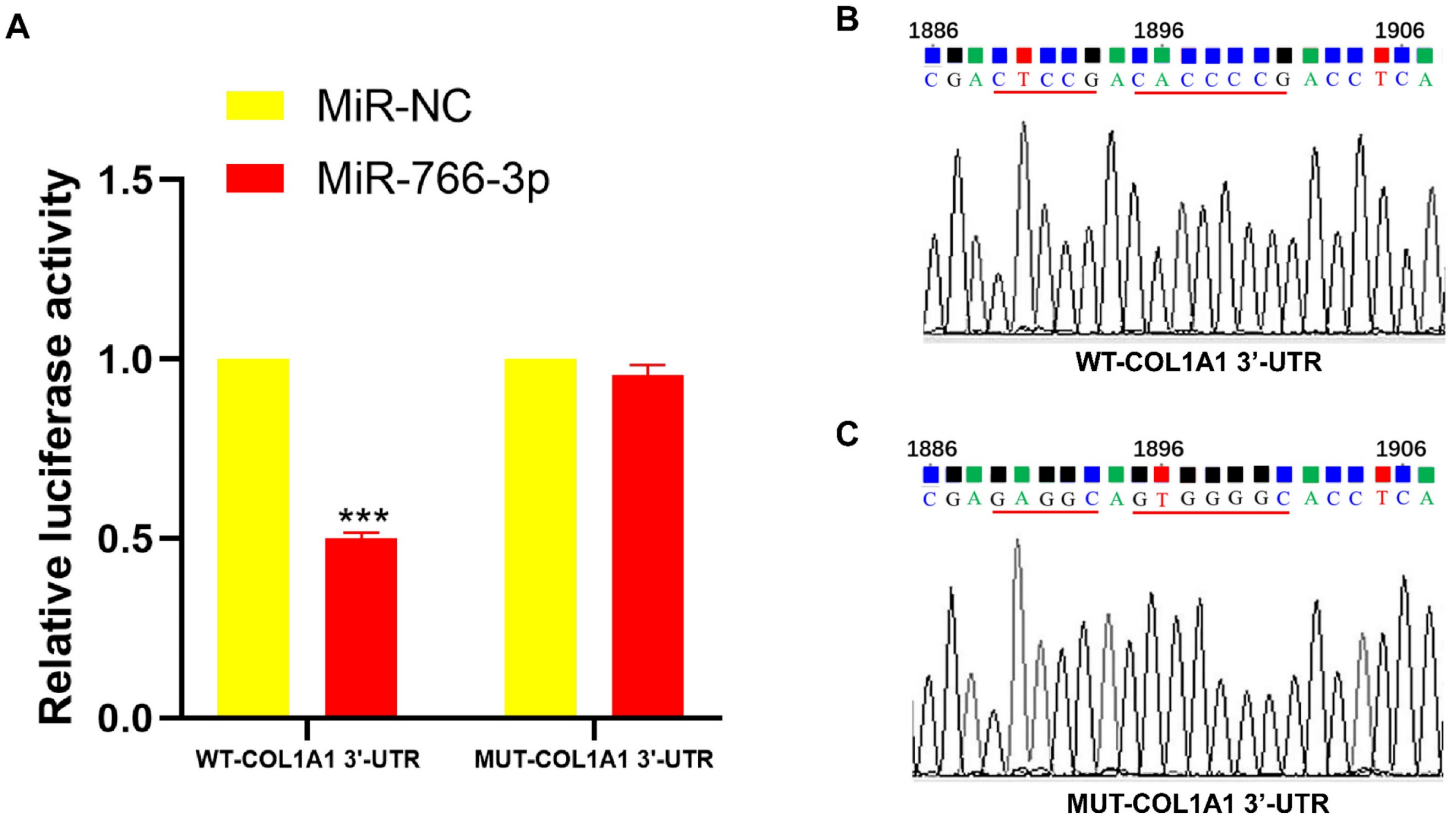
The dual-luciferase reporter assay confirmed the connection of miR-766-3p and COL1A. (A) The link between miR-766-3p and COL1A1. (B) Construction of COL1A1 wild-type plasmid. (C) Construction of COL1A1 mutant plasmids. Statistical significance is expressed as ****P < 0.0001, ***P<0.001, **P<0.01 and *P<0.05.

To verify the binding ability of miR-766-3p to COL1A1, a dual luciferase reporter assay was applied. The results showed that miR-766-3p significantly inhibited the luciferase activity of psiCHECK-2-WT-COL1A1, whereas miR-NC did not inhibit psiCHECK-2- MUT-COL1A1 (Fig. 3A-C).

### Validation core target and signal pathway by qRT-PCR and Western Blot

**Figure 4:**
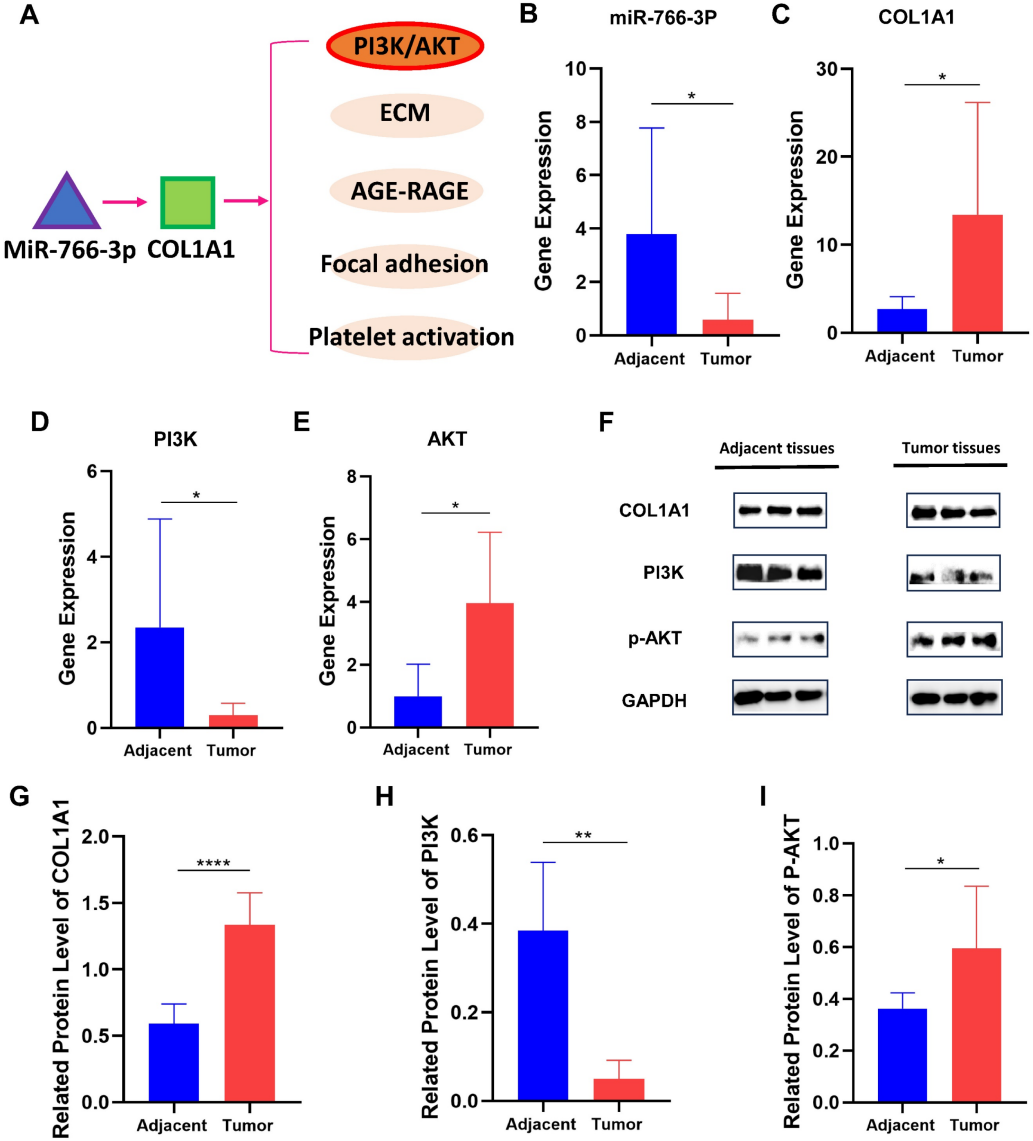
The expression levels of miR-766-3p, COL1A1 and PI3K/AKT in different stomach tumor tissues and normal gastric tissues. (A) The downstream pathway of COL1A1; (B) MiR-766-3p mRNA expression; (C) COL1A1 mRNA expression; (D) PI3K mRNA expression; (E) AKT mRNA expression; (F) COL1A1, PI3K and p-AKT protein expression in normal tissue and GC tissue; (G) The relative quantitative expression of COL1A1. (H) The relative quantitative expression of PI3K. (I) The relative quantitative expression of P-AKT. Statistical significance is expressed as ****P < 0.0001, ***P<0.001, **P<0.01 and *P<0.05.

We analyze the downstream pathway of COL1A1 by the Davide database and get many signal pathways (Fig. 4A). Of them, PI3K/AKT signal pathway is a fundamental one, with frequent oncogenic alterations in GC 20. Thus, we detected expression levels of miR-766-3p, COL1A1and PI3K/AKT of 30 patients' stomach carcinoma tissues and adjacent normal tissues via RT-qPCR and Western Blot. As a result, we found COL1A1 had a significantly higher expression level in tumor tissues than in normal ones, while miR-766-3p, conversely, had a significantly lower expression level in tumor tissues (Fig. 4B, C). Regarding the PI3K/AKT signal pathway, PI3K is low-expressed and AKT is over-expressed in gastric cancers, and there is negative regulation between them (Fig. 4D, E). At the level of protein expression as shown in Fig. 3F. COL1A1 also differs significantly between tumor and normal tissues (Fig. 4G). Relative quantitative protein expression levels of PI3K/AKT are consistent with the mRNA level (Fig. 4H, I).

## Discussion

MiRNAs as a class of regulatory factors involved in tumor regulation, and many studies show that miRNAs influence tumor progression by performing many functions, including cell division, cell differentiation, angiogenesis, migration, apoptosis and oncogenesis 21,22. For example, by directly binding to the PTEN, miR-21 may increase the proliferation, invasion, and migration of GC cells 23. And miR-21 could promote GC via activating the PI3K/AKT pathway 24. In GC cells, miR-375 is dysregulated, which promotes the growth of the PI3K/Akt pathway and cell survival 25. In our previous study, by creating the circRNA-miRNA-mRNA (CMM) network and the protein-protein interaction (PPI) network, we discovered that miR-766-3p plays a crucial role 14. However, the mechanism of miR-766-3p in GC is unclear.

In this study, the potential targets of miR-766-3p were predicted and screened by using Tarbase and Targetscan databases, and COL1A1 was identified as the core target of miR-766-3p by combining TCGA and HPA databases. The qRT-PCR and Western Blot also demonstrated that the miR-766-3p and COL1A1 affect the progression of GC. According to bioinformatic analysis, COL1A1 is a hydrophilic, negatively charged secreted protein that is crucial for the formation of collagen structures and cell adhesion 26. As we all know, COL1A1 has been found to be elevated in many cancers and affects various signal pathways, such as gastric, colorectal, breast and thyroid tumors 27-31. And miRNA-98 regulation has been demonstrated to cause lower COL1A1 mRNA levels 32. Interestingly, our study also found that miR-766-3p can downregulate the high level of COL1A1 in GC. The enrichment analysis revealed that the COL1A1 was highly correlated with PI3K/AKT signal pathway, and it promoted the activation of PI3K/AKT 33. In GC, the PI3K/AKT signal pathway plays an important role. For example, SLC1A3 through the PI3K/AKT pathway to hasten the growth of gastric cancer 34. LGR6 may accelerate the development of GC via the PI3K/AKT/mTOR pathway 35. The activation of NF-B and the PI3K/AKT/SP1 axis is necessary for the UBAP2L-induced EMT process in GC cells 36. Gastric cancer is prevented from developing and metastasizing by BFAR via the PI3K/AKT/mTOR signal pathway 37. These studies show that the PI3K/AKT signaling pathway influences the progression of GC. Furthermore, multiple pieces of evidence have demonstrated that miRNAs can regulate PI3K/Akt signal directly and partially identified the processes behind their oncogene or tumor-suppressor functions in GC. MiR-196b was found to accelerate GC tumor growth by promoting cell cycle and cell proliferation, possibly by activating the PI3K/Akt/mTOR pathway 38. On the other hand, miR-181d and miR-203 were found to reduce GC cell growth by targeting PIK3CA and, as a result, attenuated Akt activation 39. It indicates that the PI3K/AKT signal pathway is involved in the occurrence and progression of gastric cancer.

**Figure 5:**
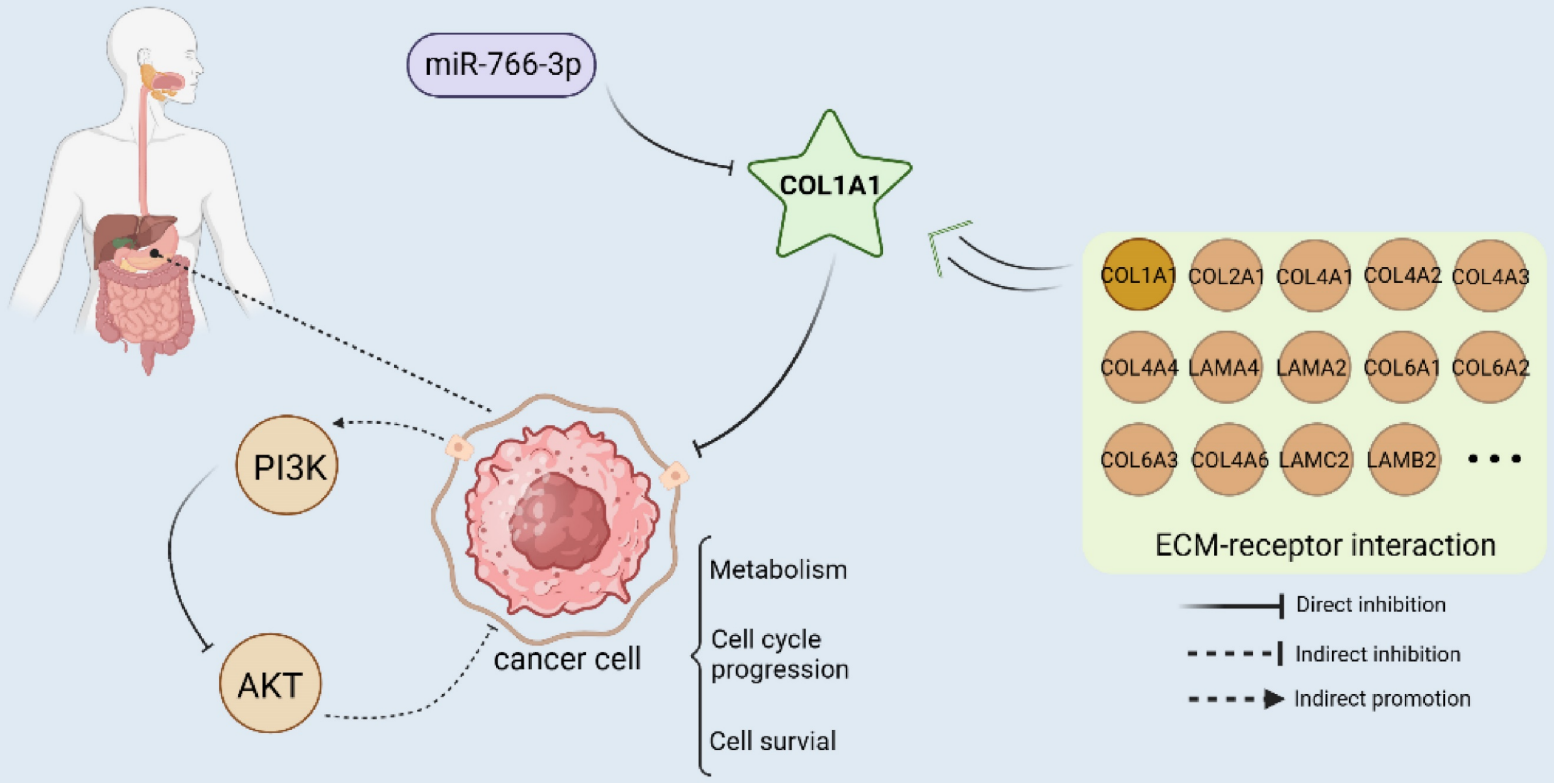
MiRNA-766-3p inhibited gastric cancer via targeting COL1A1 and regulating the PI3K/AKT signaling pathway. (Created with BioRender.com)

In summary, we demonstrate that miR-766-3p is under-expressed in the tumor tissues of gastric cancer patients. The low expression of 766 was significantly associated with advanced TNM stage, primary tumor and lymph nodes (Fig. S1, Table S2). COL1A1 is an important target of miR-766-3p and is inversely correlated with the expression of miR-766-3p. The luciferase Assay indicated that miR-766-3p suppresses gastric cancer through directly targeting the 3'-UTR of COL1A1 mRNA and down-regulating the levels of COL1A1. It can also participate in the progression of gastric cancer by influencing the PI3K/AKT signal pathway. Meanwhile, COL1A1 is one of the main components of the extracellular matrix (ECM). ECM is also the key component of the tumor micro-environment (TME), which has complementary effects on the development and metastasis of tumors in diverse ways 40. MiR-766-3p/COL1A1/PI3K/AKT may inhibit gastric cancer progression through suppressing ECM, metabolism, cell cycle progression and cell survival (Fig. 5). Our findings suggest a potential molecular basis for the genesis and progression of gastric cancer, which may lead to new approaches to diagnostics and treatment. In the future, it may represent a potential treatment strategy against the GC.

## Supplementary Material

Supplementary figure and tables.

## Figures and Tables

**Table 1:** Patient characteristics (n=30)

	n (%)
**Sex**	
Male	18 (60)
Female	12 (40)
**Age**	
>70	4 (13)
50-70	21 (70)
<50	5 (17)
**Location**	
Lower third	19 (63)
Middle third	6 (20)
Upper third	5 (17)
**Stage**	
Ⅰ	10 (33)
Ⅱ	8 (27)
Ⅲ	8 (27)
Ⅳ	4 (13)
**T**	
T_1_	10 (33)
T_2_	4 (13)
T_3_	7 (24)
T_4_	9 (30)
**N**	
N_0_	11 (37)
N_1_	11 (37)
N_2_	2 (6)
N_3_	3 (10)
N_x_	3 (10)
**M**	
M_0_	25 (83)
M_1_	2 (6)
M_x_	3 (10)

**Table 2:** The primer sequences obtained from the NCBI database

Gene name	Forward primer (5′- 3′)	Reverse primer (5′- 3′)
miR-766-3p	CGACTCCAGCCCCACAGC	AGTGCAGGGTCCGAGGTATT
COL1A1	TCGGAGGAGAGTCAGGAA	ACACAAGGAACAGAACAGTC
GAPDH	CCTGCCTCTACTGGCGCTGC	GCAGTGGGGACACGGAAGGC
U6	CTCGCTTCGGCAGAACA	ACGCTTCACGAATTTGCGT
PIK3CA	CCTGCTTTTGGAGTCCTATTGT	ATCTGGTCGCCTCATTTGC
AKT1	CTCTTTCCAGACCCACGACC	ACAGGTGGAAGAACAGCTCG

**Table 3:** The distributions of potential hub mRNA from the network

Cluster	Hub miRNA	Degree	Expression	P-value	Status
1	THBS2	9	0.5	8.06E-06	Up
2	COL1A1	8	0.6	5.56E-05	Up
3	FGG	6	0.9	0.003	Down
4	FGB	5	0.6	0.014	Down
5	PLAU	5	0.5	0.001	Up

## Abbreviations

GC: gastric cancer
miRNA: microRNA
DEGs: differential genes
TCGA: The Cancer Genome Atlas
GTEx: Genotype-Tissue Expression
HPA: Human Protein Atlas
CMM: circRNA-miRNA-mRNA
ECM: extracellular matrix
TME: tumor micro environment
