# Supplementary material for: MiRNA-766-3p inhibits gastric cancer via targeting COL1A1 and regulating PI3K/AKT signaling pathway
Source: J Cancer. 2024 Jan 1;15(4):990–8. doi: 10.7150/jca.90321 (PMC10788715; doi:10.7150/jca.90321)
Supplement: Supplementary file 1 — Supplementary figure and tables. [file jcav15p0990s1.pdf]

Supplementary Figure 1.

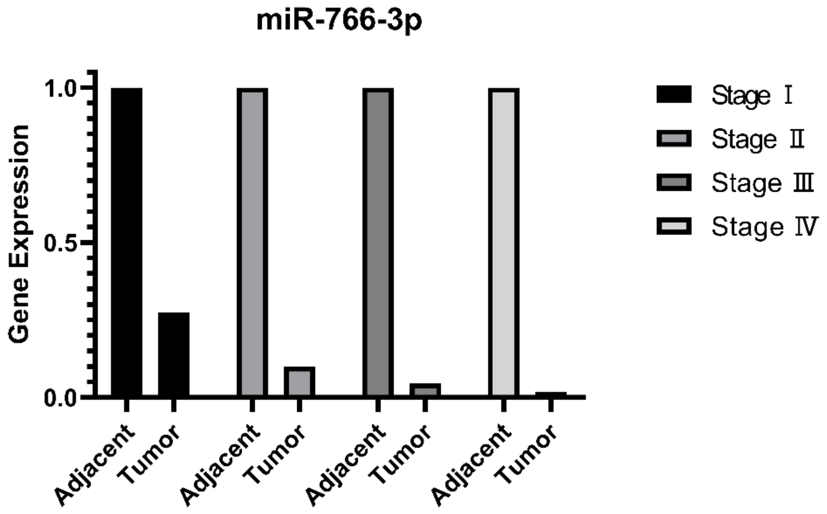

Supplementary Tables

Supplementary Table 1. Age factor between clinical sample and validation dataset

Age of clinical sample	Age of validation dataset	p value
63, 66, 48, 72, 49, 83, 65, 65, 67, 42, 70, 70, 72, 62, 65, 69, 68, 66, 65, 66, 70, 78, 62, 59, 58, 50, 49, 58, 64, 69	68, 59, 56, 62, 42, 79, 54, 48	$p > 0.05$ ($P = 0.1838$)

Supplementary Table 2. Correlation between expression of MiR-766-3p and clinicopathological characteristics of patients with gastric cancer

Characteristics	Case(n)	MiR-766-3p		p Value
		High (n)	Low (n)	
**Age (years)**				
≤60	22	12	10	0.4090
> 60	8	3	5	
**Gender**				
Male	18	10	11	0.8953
Female	12	6	6	
**TNM stage**				
I-II	18	11	7	0.1360*
III-IV	12	4	8	
**Primary tumor**				
T1-T2	14	12	2	0.0003*
T3-T4	16	3	13	
**Lymph nodes**				
N0-N1	22	19	3	0.0437*
N2-N3	5	1	4	
